# The impact of the student perception of teacher talk on student language learning attainment

**DOI:** 10.1186/s40359-025-02508-3

**Published:** 2025-03-09

**Authors:** Peihong Wu, Zhimin Wang

**Affiliations:** 1 School of Cultural Tourism and Health, Shanxi Institute of Science and Technology, Jincheng, 048000 Shanxi China; 2 School of Foreign Languages, Taiyuan University of Science and Technology, Taiyuan, 030024 Shanxi China

**Keywords:** Student perception of teacher talk, Enjoyment, Emotional exhaustion, Self-efficacy, English language learning attainment

## Abstract

While prior studies provide valuable insights into the role of teacher talk and its impact on learning outcomes, there remains a need for further research on how contextual factors may interact with student perceptions to influence language learning. This study explores how students’ perceptions of teacher talk influence their English language learning outcomes, focusing on the mediating roles of student enjoyment and emotional exhaustion, as well as the moderating role of self-efficacy. The research involved a cross-sectional survey with 357 university students, and the data were analyzed using structural equation modeling (SEM). Findings affirm that mindful perceptions of teacher talk enhance students’ enjoyment level thus the achievement of better English language learning. On the other hand, negative perceptions result in a condition known as the emotional exhaustion which has negative influence on learning experience. Contrasting with emotional exhaustion, student self-efficacy was established to moderate interaction with enjoyment and increase the impact of advantage on language attainment. Such results emphasize the necessity of constructing positive and stimulating teacher-student communication as well as the enhancement of student’s perceived self-efficacy.

## Introduction

The ability to convey oneself in the English language is highly valued in the contemporary world, where learners from diverse backgrounds often interact in academic, professional, and personal domains [1, 2]. In formal educational settings, the central role of teachers in facilitating and guiding language use is undeniable [3]. Among the myriad factors influencing second language acquisition, teacher talk, defined as the language teachers use during instruction, has emerged as a particularly salient determinant of students’ performance [4]. Previous studies suggest that the quality and quantity of teacher talk can shape learners’ cognitive and affective engagement [5], and that students’ reception of this talk, how comprehensible or meaningful they perceive it, strongly influences motivation and active participation [5, 6]. Conversely, negative perceptions, such as boredom or frustration, may lead to disengagement and hinder language development [7, 8].

Despite the evident significance of student perception of teacher talk, prior research has not fully explored how and why these perceptions translate into differential learning outcomes in second language contexts. For example, scholars have noted that it is unclear how teacher talk translates into concrete student learning outcomes [5, 9]. As such, analyzing student perception of teacher talk as a concept, may help its role in determining different aspects of language learning may be profound [10]. Thus, studying the factors which can either enhance or reduce the correlation between the specified perception and interest in the outcome of language learning could be helpful in improving educational practices, as well as in fine-tuning learning arrangements.

Moreover, there remains a gap in understanding the mediating and moderating mechanisms that underpin this relationship. Scholars have highlighted two key affective processes, student enjoyment and emotional exhaustion, as potentially critical mediators [11, 12]. Enjoyment, characterized by the joy and personal satisfaction derived from engagement in learning tasks, consistently predicts academic performance across various domains, including language learning [13, 14]. Conversely, emotional exhaustion, feelings of fatigue and withdrawal, can detract from a student’s capacity to manage learning efforts and persist in challenging tasks [15, 16]. The knowledge of the processes which connect student perception of teacher talk and English language learning outcomes is crucial in designing instructors’ strategies that foster positive learning and avoid negative occurrences.

In addition, the moderating variable of student self-efficacy cannot be left out in this relationship as well. Self-efficacy according to Bandura [17] and Ali, Wang [18], entails the confidence of people in their ability to perform specific task or actions required for accomplishing specified levels of performances. Various aspects of the model can be best defined by illustrating its theoretical constructs in the context of students’ perceptions of their own English language learning capacities and how they affect students’ motivation, participation, persistence, and learning [19]. Hence, understanding how student self-efficacy can moderate the relationships between the student perception of teacher talk and English language learning attainment, one can extend some more light on the conditions that promote extraverted teacher talk for the improvement of language proficiency of students.

**Fig. 1:**
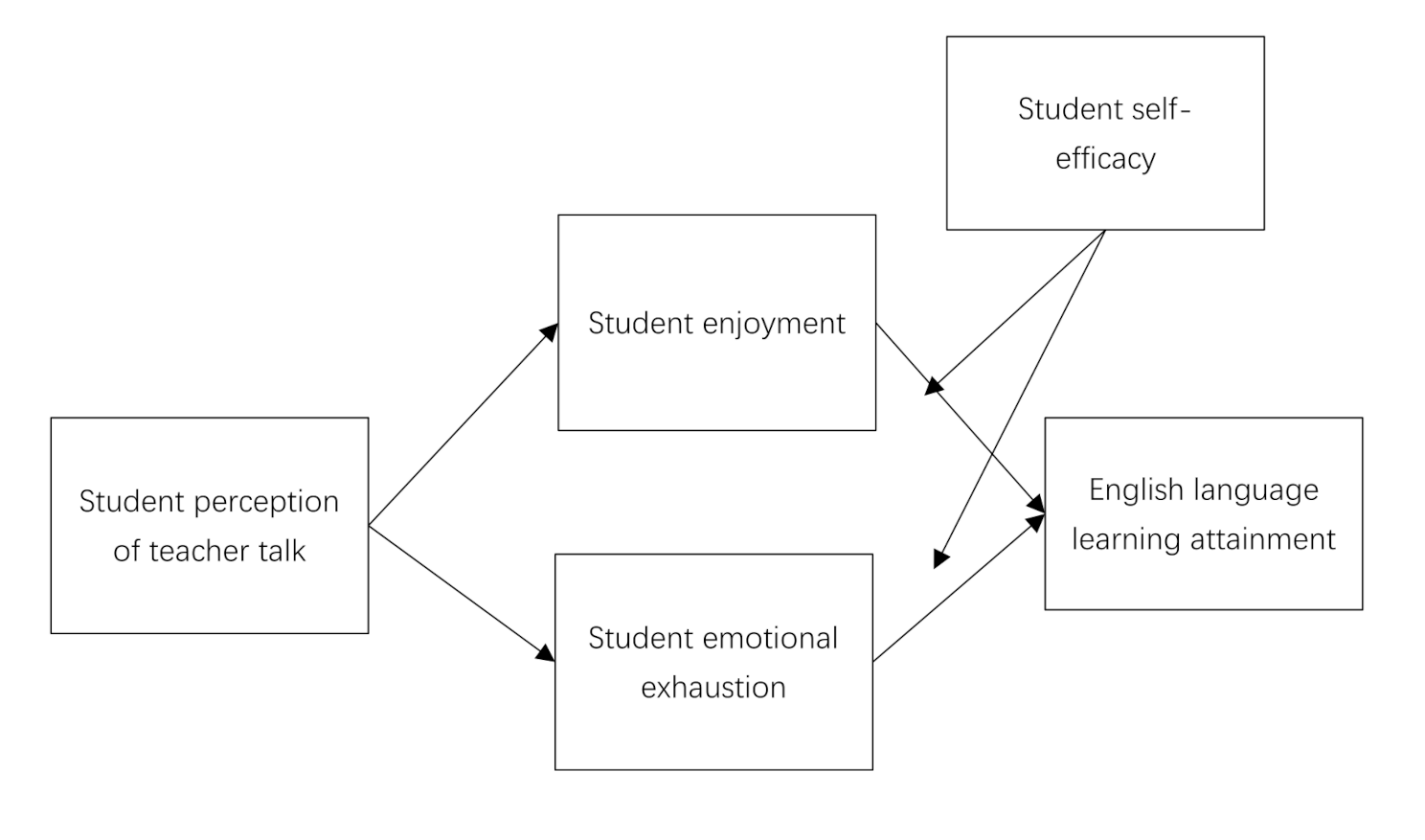
Proposed model

To address above gaps in the literature, this uses following research question to develops a model (Fig. 1) to investigate how students’ perceptions of teacher talk influence English language attainment through the mediating roles of student enjoyment and emotional exhaustion, while also examining whether self-efficacy strengthens or weakens these pathways.

1. How do students’ perceptions of teacher talk influence their English language learning outcomes, particularly within technology-mediated instructional contexts?
2. To what extent do students’ affective states, enjoyment and emotional exhaustion, mediate the relationship between perceived teacher talk and language-learning performance?
3. Does self-efficacy moderate these mediated pathways?

By integrating these affective and psychological constructs into a unified framework, this study aims to provide a more nuanced understanding of the conditions under which teacher talk is most beneficial—or detrimental—for second language learners. Ultimately, these insights can guide educators in tailoring their instructional language and scaffolding strategies to optimize student motivation, reduce burnout, and enhance overall language proficiency.

## Literature review and hypothesis development

### Student perception of teacher talk and english language learning attainment relationship

This paper shows that student attitude towards teacher talk is a key factor that affect students’ performance, participation, and motivation in their learning of English as a foreign language. Teacher talk therefore refers to the total of all the verbal responses from the teacher during learning intervention that entails; teacher explanation, teacher feedback, and interactional discourse [20, 21]. Teacher talk refers to the extent, quality, and frequency of teacher’s speaking in the classroom, which is essential as it offers the major source of input to learn second language by students [22]. Nonetheless, it is equally important to realize that the affluent use of language that we have been discussing as a part of teacher talk is not just a question of the quality of language being employed. Observations of teacher-student communication, specifically, students’ attitudes of perceiving teacher talk as comprehensible, reassuring, and inviting, lead to increased participation, which in turn results in greater language learning [23]. On the other hand, negative perceptions like perceiving teacher talk as difficult and complex may have negative contribution towards student motivation and advancement in English language learning [24].

Intuitively, existing studies have established a correlation between the type of teacher talk from the students’ perspective and their language learning progress [5, 10, 25]. For example, Mercer and Dörnyei [25] argue that when students are able to understand teacher talk and consider the talk to be scaffolded, students feel competent and autonomous in the learning process, which are features of intrinsic motivation according to self-determination theory by Deci and Ryan [26]. This feeling of efficiency and independency results in better cognitive absorption and longer time spent on accomplishing language acquisition goals, which ultimately leads to a higher level of English language attainment [2, 27]. Research has also indicated that teacher talk in terms of use of open-ended questions, urges to expand and generate more language use, and generic prompts that allow students to take over the conversation, enhances students’ language development by involving them actively in language use and critical thinking [28, 29]. These interactional properties of teacher talk are not only beneficial in offering complex forms of input but also in contributing to the development of a stimulating social context that enables learners to engage in repeated acts of target language use.

The above findings are also supported by other studies that establish the connection between student perceptions of teacher talk and English language learning based on interactive and student-oriented teacher talk. Kim and Elder [30], identified that in a context where teacher talk is used in conjunction with interactive techniques such as elicitation, scaffolding, and feedback, higher levels of learning and language attainment were achieved. If the students are able to come to the conclusion that the teacher talk is not a one way form of communication but actually engages with student needs, then they will be encouraged to learn through the more meaningful form of communication which is language acquisition [31, 32]. This is in concord with the hypothesis by Long [33], which suggests that language learner gains from interactional modifications in teacher talk that makes input understandable and that which offers the learner an avenue to respond to. Thus, it is significant for the students’ linguistic acquisition and achievement that these aspects of the teacher’s discourse should be valued by the students.

### Mediating roles of student enjoyment student emotional exhaustion

The mediating influence of a variable, in this case, student enjoyment is critical in the interdependence between teacher talk and the performance of students in English language learning as their second language. Enjoyment, defined as affective states of pleasure, interest and intrinsic motivation in the context of learning activities constitutes one of the affective variables which can positively impact cognition and academic achievement [14]. Fredricks, Blumenfeld [34], argue that when student’s perceive teacher’s talk as relevant and encouraging, this lead to their enjoyment thus creating increased commitment towards learning efforts. It also entails improved mastery and usage of English language amongst the beneficiaries owing to the enhanced engagement. Besides, enjoyment has a self-reinforcing effect; increased interest in the learning activities leads to more effort and perseverance [35], which forms the basis of becoming a second-language user [36]. Therefore, teacher talk, being perceived enjoyable mediates the level of learning and development in realizing higher level of language proficiency.

On the other hand, the level of the student burnout could explain the degree of the detrimental effects of the excessive teacher talk on the English language learning achievements [37]. A more specific aspect of burnout that needs to be surveyed is the measure of emotional exhaustion, defined as a depletion of emotional resources owing to prolonged stress and strain [11]. Thus, when in educational context the teacher’s discourse can may decrease the feelings of emotional distress among learners [15]. Scholars have found that social contexts shape emotional exhaustion [38–40]. Such a state of exhaustion can be said to have a negative impact on the students’ learning ability in terms of their ability to learn and assimilate new information, a crucial aspect in language learning [41]. In a separate study conducted by Zhang and Sapp [42], it was also evident that high levels of emotional exhaustion are inversely proportional to the levels of the students’ academic achievement because most of the exhausted learners rarely attend classes or submit assignments. Thus, by serving as a mediator variable, emotional exhaustion suggests that appropriate course mechanics may alleviate the benefits of teacher talk, an indication of a need for moderation and a favorable classroom climate.

*Hypotheses 1: Student enjoyment mediates the relationship between student perception of teacher talk and student English language learning attainment.*

*Hypotheses 2: Student emotional exhaustion mediates the relationship between student perception of teacher talk and student English language learning attainment.*

### Moderating role of student self-efficacy

Another important research question is whether and how student self-efficacy moderates the impact of teacher talk on second language learning and more specifically, English language learning [43–45]. Self-efficacies, which refers to students’ expectations regarding their capabilities for learning and achieving academically [17], can have a strong impact on the form through which students comprehend as well as their reaction towards teacher’s discourse [46]. Self-efficacy has been found to influence students’ approach towards tasks as depicted by the fact that highly efficacious students tend to believe that they can accomplish difficult tasks and are more likely to persevere when faced with obstacles [19]. Applying the framework of self-efficacy in language acquisition context, it can be suggested that high level of self-efficacy can support the efficiency of teacher talk by increasing students’ confidence in the future learning process and in the ability to understand and use the target language. For example, Graham [47], notes that self-efficacious students are more capable in using the teachers’ feedback as well as instructional talk to enhance language development, in part because they will be more inclined and persistent in their practice unlike others who give up easily. Consequently, self-efficacy has a multiplicative effect whereby it enhances the contribution of teacher talk towards the learners’ language achievement.

However, the students with low self-efficacy may have different perceptions towards teacher talk and as such the results may be less effective. Low self-efficacy as found by Schunk and Pajares [48], causes anxiety among the students and makes them doubt their capability to do language tasks hence developing fear and reluctance not to engage in the learning process. Such students may find it difficult to engage with places where they experience teacher talk which they consider to be hard or which they consider in some way taxing and will therefore refrain from fully participating in classroom discussions or engaging in practice that involves the use of language. This in turn can lead to a vicious cycle by which low self efficacy reduces the effects of teacher talk lowering in turn the linguistic competency. In their study of the inter related mechanisms of selves, Hassankhani, Mohajjel Aghdam [49], also noted that self-efficacy affected not only motivation but also moderated the consequences between instructional practice and performance, these findings imply that enhancing self-efficacy could help optimize the outcomes of teacher talk in language learning context.

In addition, the analysis reveals that self-efficacy can operate as a moderator of the relationship between instructional practices and student achievement [50], thus implying potential instructional approaches to be used when teaching to a heterogeneous class of students. The studies have focused on how students with different levels of self-efficacy study during teachers’ talk time and found that there is a difference in students’ study behavior on the basis of their self-efficacy [51, 52]. For instance, Tschannen-Moran and Hoy [53], offer a study that indicates how teachers can leverage self-efficacies through mastery experiences, positive verbal persuasion and coaching or role modeling in an effort to enhance the learning environment. It is also explored by Goddard, Tschannen-Moran [54], that in a practical activity, this could include offering gradual increases in difficulty to help build up student’s confidence, using affirming language and feedback, and modelling the correct use of language. Moreover, it has been reported by Kim and Baylor [55], that presence of adaptive learning/teaching approaches that incorporates learners’ self-efficacy levels contributes towards better learning engagement and achievement. Furthermore, the integration of these strategies will help offset the drawbacks involving low self-efficacy and enhance the advantages of teachers’ instructional talk so as to achieve better English language mastery for students with different learning characteristics.

#### Hypothesis 3

*Student self-efficacy moderates the indirect effect of student perception of teacher talk and student English language learning attainment via student enjoyment such that the indirect is more positive at a high level of student self-efficacy than at low level of student self-efficacy.*

#### Hypothesis 4

*Student self-efficacy moderates the indirect effect of student perception of teacher talk and student English language learning attainment via student emotional exhaustion such that the indirect is less negative at a high level of student self-efficacy than at low level of student self-efficacy.*

### The present study

The present study uses an elaborate model that captures multiple variables to determine the relationship between student’s perception of teacher’s talk and English language learning attainment with mediating and moderating effects. Teacher talk specifically refers to the student perception of teachers’ verbal input, as defined by Ellis [20], as a key that would unlock the impact of student perceptions of teacher talk on the English language learning outcomes. This relationship is hypothesized to be mediated by two critical affective variables: students’ enjoyment and students’ emotional exhaustion. Enjoyment, defined as positive affect and intrinsic motivation during learning activities as described by Pekrun, Goetz [8], will help increase cognitive investment and, on this ground, impact English language learning attainment. Emotional exhaustion, one of the sub dimension of burnout emphasizing on the feeling of fatigue and decrease in engagement Maslach, Schaufeli [11], is expected to reduce students’ capacity to capture and store new linguistic information, thereby negatively influencing second language acquisition. Apart from these mediating variables, there is another variable, which is student self-efficacy as the moderation variable in our proposed model (Figure: 1). Self-efficacy which refers to the level of confidence learners hold to persevere in language learning tasks [17], is assumed to determine how students interpret and/or respond to teacher talk. By hypothesizing a positive, moderational relationship between self-efficacy and the teacher explanation variable, it is assumed that the use of supportive teacher talk will be more beneficial and effective for self-efficacious students than less self-efficacious students because these kinds of students are more capable to work hard on challenging tasks. On the other hand, low self-efficacy might enhance the detrimental effects of critical or unsupportive teacher talk which on pupils’ level results in increased anxiety and subsequent avoidance of learning [56]. Through the incorporation of these mediating and moderating variables into the flowchart, a key aspect of the design of this proposed model is to depict the temporal and contextual complexity of language learning to give a comprehensive explanation of the factors that determine English language learning. This is a broad-spectrum strategy intended to be beneficial for educators, presenting the need to embrace both emotions and thought processes when designing instruction methods [25, 57].

The present study mainly contributes to the literature by answering the following research questions: (1) to what extent is student-perceived teacher talk an influential factor within attaining better English language learning through the mediating variable of student enjoyment? This notion is in line with a study by Pekrun, Goetz [14], whereas Dewaele and MacIntyre [36], also found out that, when the students considered teacher talk as comprehensible, motivating, and affiliative, their enjoyment and self-generated interest would be increased, thereby promoting foreign language learning. Intrinsic motivation mechanisms indicate enjoyment and suggests that positive affective experiences play a key function in enhancing detailed cognitive processing and higher persistence in undertaking language learning activities [34]. (2) To what extent student perceived teacher talk affects English language learning attainment through the mediator variable, namely student emotional exhaustion. Thus, by the end of a term, victims of academic stress may experience a state when they feel emotionally detached from their course of study and suffering from burnout and therefore, when handling a new language, the students would be impaired in their ability to retrieve information as their cognitive processes have been compromised. When the teacher talk is seen negatively it is likely to increase the feelings of emotional exhaustion and hence discourage language learning [42]. (3) To what extent does student self-efficacy have a moderation effect on the student’s enjoyment and emotional exhaustion belief towards English language learning achievement? It is important to note that self-efficacy refers to students’ beliefs with regard to their capabilities regarding the accomplishment of given learning tasks in language, such that enjoyment can enhance the former while minimizing the latter in a given setting [19]. Self-efficacy improves students’ stability and enthusiasm since it helps shield one from adverse stress impacts and consistently supports more effective academic outcomes [48].

## Methods

### Data collection procedures

The participants of the study consisted of 357 Chinese learners of English drawn from a school district in a Chinese city through convenience sampling. A school district in a Chinese city may have been chosen because it is readily accessible to the researchers. A researchers already be in the region and have existing relationships with the schools, making it easier to obtain permission and recruit participants. In addition, author have chosen a district that has a higher concentration of schools with English language programs, increasing the likelihood of gathering rich, relevant data for the study Author has used convenience sampling method for this study for several reasons. First, convenience sampling is cost-effective because it saves time and resources. Second, convenience sampling helps researchers make use of whatever participants are available. For example, in small, localized studies or when studying specific groups of individuals who are readily accessible (e.g., university students for campus studies), it may be the most feasible option. Prior scholars also observed that convenience sampling can be particularly useful for testing hypotheses or conducting pilot studies before larger-scale, more rigorous sampling methods are employed. In Structural Equation Modeling (SEM), determining an appropriate sample size is essential for conducting a reliable and efficient analysis. To ensure that the sample size was sufficient, we used statistical power analysis to calculate the required number of participants, utilizing the G*Power software. Statistical power is a critical measure that indicates the likelihood of detecting an effect if one truly exists, and it is important for ensuring that the results are not due to random chance. For this study, statistical power of 0.95, which exceeds the commonly recommended threshold of 0.80, which provides confidence in the robustness of the results [58, 59]. By surpassing this threshold, the sample size used in this study is more than adequate for SEM analysis, reducing the risk of Type II errors and increasing the reliability of the findings. As a result, the sample size employed in this study is deemed sufficient to perform SEM analysis effectively, ensuring the statistical rigor and accuracy of the model estimation. The participants were university students in their first-year English preparatory program to enable them do majors that utilize English language as their medium of instruction. The course followed the guidelines of the Common European Framework of Reference (CEFR) for languages, particularly, it can be attributed to the Learning, Teaching, and Assessment, mentioned by West, Güven [60]. The CEFR is intended for use in describing, comparing and coordinating the provision of language education across Europe and divides the ability to use a language into six broad levels (A1-C2). From 357, there were 196 males and 161 females with an average age of 31 to 40 years. The demographic characteristics of participants are shown in Table 1.

**Table 1:** Demographic information of the samples

Variables	*N*	Percentage	Variables	*N*	Percentage
**Gender**			**Education Level**		
Male	196	54.900	High School or below	88	24.600
Female	161	45.100	College or Diploma	64	17.900
**Age**			Graduate	93	26.100
Between 18–25 years	100	28.00	Masters or Above	112	31.400
Between 26–30 years	88	24.600			
Between 31–40 years	96	26.900			
> 40 year old	73	20.400			

### Instruments

#### Student perception of teacher talk

Perceived teacher talk was measured **by six items** specifically developed for this study, stemming from prior research on academically productive types of teacher talk [5, 61–63]. Consequently, while many studies over the years have captured the characteristics of classroom discourse from the teacher’s vantage point, no prior research has targeted the perceptions of students as the main protagonists of the analysis. This study however developed and pilot tested the student-perceived teacher classroom talk scale to assess the perceptions that students have on their English language teacher’s classroom talk. The scale aligns with four key goals for fostering classroom discussions: It assists in the following ways: (1) sharing of ideas among the students, (2) active listening amongst the students, (3) builds the students’ critical thinking skills, and (4) cooperative thinking [61, 62]. Students were also urged to indicate the frequency with which their English teachers integrated academic talk based on six items (e.g., “My English language teacher makes us explain the ideas in the classroom,” “My English language teacher permits adequate time for responding to questions in the classroom,” “My English language teacher makes us assess one another’s ideas in the classroom”). The responses were collected using a four-point Likert scale where 1 represented rarely, and 5 represented frequently. A higher value for the sum of scores was an increase of the academically productive talk according to the students’ perception.

#### Students’ emotions in class

To assess the students’ self-reported emotions during the class, we created a scale borrowed from the class-related part of the AEQ [8]. To measure the extent to which students find attending English language class enjoyable, the following four items were used (e.g., “I often look forward to my English language class” and “I enjoy going for my English language class”). It was on a Likert scale with response choices running from 1 (strongly disagree) to 5 (strongly agree). A higher score on the subscale were suggested to result in greater enjoyment.

The level of student emotional exhaustion was assessed with the help of Maslach Burnout Inventory-Student Survey (MBI-SS) [11], and respectively the subscale of emotional exhaustion. Based on the Maslach Burnout Inventory, MBI-SS we used to assess burnout phenomena in academic context, the signs of which are fatigue, and depletion of emotional reserves. This is a **four-item** subscale that that has been used in many studies in education setting and is known for its ability to ascertain reliably the level of students’ emotional exhaustion [64]. Some of the sample items that were used: “I feel emotionally drained by my studies”; “I feel burned out from my studies.” The Likert scale used for measuring these variables ranged from 1 (never) to 5 (always). The use of the MBI-SS in our study enabled the measurement of levels of students’ emotional exhaustion as influenced by perceived teacher talk and their resultant.

#### Questionnaire of self-efficacy in learning a foreign Language (QSLL)

The QSLL is an **11-items** structured scale applicable to gauge the participants’ perceived self-efficacy towards the learning of English as foreign language [65]. This instrument comprises a single overarching factor representing overall self-efficacy in English as a second language, as well as two specific group factors: Reading/Listening self-efficacy and Speaking /Writing self-efficacy. The questionnaire items are based on the Common European Framework of Reference for Languages: Language learning, language teaching, language assessment [66], which is the official document defining the reference levels for communicating in a foreign language. An example item for listening is, “I can watch and understand English films and TV series without English/Chinese subtitles.” The responses were on a Likert scale; 1– strongly disagree, 5- strongly agree. The scores were generally derived from the scale and the evaluation criteria were used to calculate a mean score with the higher score relating to higher self-efficacy.

#### English language attainment

Learner performance was measured in terms of participants’ average English examination result obtained at the end of the English preparatory program. These scores were self- generated by the universities as a composite of bookmark quizzes, mid-semester tests, and semester-end tests. One of the most significant decisions was the choice of the exam structure which consisted in adopting the CEFR as the common framework for the examination. Read, write, listen, and the speak skills were tested, and the scores obtained by learners were summed to give a total score. The maximum that could have been scored in this project was 100%. These examinations were developed and conducted by an independent testing office in each university, mainly the English as foreign language teachers were involved in the conception, writing, and execution of the exams during the academic year. The examinations were internally double marked according to guidelines from the testing offices. The two independent markers separately assigned grades to each student’s portfolio, but in case of differences, the markers discussed the matter with a moderator with the common agreement on the final grade.

## Data analysis and results

### Analytical strategies

This study was conducted in compliance with the recommendations highlighted by Kumar, Upadhyay [67], and the six stages of data analysis were included in this study are enumerated as follows: First, the missing data, outliers, and multicollinearity were examined by the author. To eliminate the possibility of common method variance, different tests were conducted in the second stage. In the third phase, confirmatory factor analysis (CFA) was conducted for all the measures to establish the fit indices, internal consistency, and validity coefficients of the study. The fourth stage involved assessing the model fit values of the research model as compared to the other models. Based on the guidelines of Prügl and Spitzley [68], SEM analysis was used on AMOS in the fifth stage to assess the direct effects of the variables, whilst the control variables were regressed on English language learning on English language learning attainment. Furthermore, Hayes [69] PROCESS Model 4 macro was used to establish mediation effects of student enjoyment and student emotional exhaustion. Finally, the moderated mediation analysis was conducted using Hayes [69] PROCESS Model 7. Whereas for Model 4 and model 7, a simple random sample of size B = 5000 was chosen and using 95% level of confidence interval in order to increase the accuracy of the bootstrap estimation [69].

### Measurement model

In order to assess the reliability, validity of all the constructs and, thus confirm the quality of the measurement model we conducted reliability analysis, convergent validity and discriminant validity test. For convergent validity, scholars proposed that loading scores should be at least 0.60, while loading values higher than 0. 70 is perfect for the analysis of hypothesis [70]. Further, to assess the internal consistency and reliability of the measures, the CR and CA coefficients were calculated. Research results revealed that both coefficients, namely CR and CA were above 0. 70, as shown in Table 2, which indicates an acceptable level of reliability [70]. Moreover, as presented in Table 2, all the constructs possessed the AVE values more than 0.50 [58], indicating an adequate level of convergent validity. Therefore, the results suggested that the composite reliability values reached the criterion for internal consistency and reliability. Consequently, it was found that all the constructs of our measurement model had acceptable levels of reliability and convergent validity.

**Table 2:** Results of confirmatory factor analysis

Construct	Items	CA	CR	AVE
Student perception of teacher talk	6	0.942	0.950	0.760
Student enjoyment	4	0.825	0.870	0.630
Student emotional exhaustion	4	0.773	0.830	0.560
Student self-efficacy	11	0.958	0.960	0.690
Listening	5	0.786	0.840	0.520
Writing	6	0.820	0.830	0.530
Reading	6	0.821	0.870	0.520
Speaking	5	0.870	0.900	0.640
Testing	6	0.868	0.890	0.590

Note: CA = Cronbach’s alpha; CR = composite reliability; AVE = average variance extracted

Furthermore, discriminant validity of all the measurement items was confirmed through a multi-method approach. Initially, we scrutinized the cross-loadings of the items of all constructs, as detailed in Table 3. From Table 3, it is apparent that all instruments exhibit loadings greater than 0.60, with substantial loadings in their designated positions and minimal loadings in other columns [71]. Secondly, we analyzed the square root of the AVE of each construct that was depicted in Table 4 against the correlation coefficients between the constructs. From these comparisons, it becomes evident that all the AVE square roots exceed the correlation coefficients as proposed by [70]. Finally, we employed CFA model fit approaches to evaluate the discriminant validity of the research model in the current research [72]. Table 5 provides a comparison of all CFA models demonstrates that all models’ χ2 values exceed the recommended thresholds, indicating significant differences among the models. Furthermore, all CFI differences were above the cutoff value of 0.002. Consequently, discriminant validity is confirmed through both the χ2 difference and CFI difference criteria.

**Table 3:** Cross-loading

Constructs	Items	STSEF	SPTT	TST	SPK	RED	WRT	SENJ	LST	STEX
Student self-efficacy (STSEF)	STSEF01	**0.885**	− 0.066	0.055	0.037	0.002	0.023	0.032	0.016	− 0.038
	STSEF02	**0.880**	− 0.078	0.044	0.035	0.059	0.045	0.030	0.045	0.027
	STSEF03	**0.865**	0.005	0.046	0.033	0.029	− 0.011	− 0.012	− 0.009	0.050
	STSEF04	**0.856**	0.040	− 0.076	− 0.034	− 0.005	0.002	0.041	− 0.039	− 0.009
	STSEF05	**0.853**	0.004	− 0.013	− 0.013	0.018	0.038	− 0.010	− 0.018	− 0.019
	STSEF06	**0.848**	0.026	− 0.015	0.000	− 0.010	− 0.025	0.000	− 0.085	− 0.086
	STSEF07	**0.821**	− 0.072	0.030	0.024	− 0.023	0.002	0.008	0.035	− 0.009
	STSEF08	**0.816**	0.094	− 0.026	− 0.064	− 0.049	− 0.009	0.012	− 0.046	− 0.031
	STSEF09	**0.792**	0.011	− 0.023	0.043	− 0.059	0.031	− 0.045	− 0.028	− 0.039
	STSEF10	**0.757**	0.015	0.033	− 0.026	− 0.052	− 0.027	− 0.079	0.056	0.030
	STSEF11	**0.694**	0.053	− 0.015	− 0.054	0.063	− 0.058	− 0.021	0.122	0.129
Student perception of teacher talk (SPTT)	SPTT01	0.017	**0.958**	0.027	− 0.023	0.044	− 0.009	− 0.004	− 0.022	0.068
	SPTT02	0.026	**0.948**	0.034	− 0.022	0.036	− 0.007	− 0.009	− 0.023	0.044
	SPTT03	0.000	**0.947**	0.051	− 0.013	0.015	− 0.023	− 0.011	− 0.019	0.046
	SPTT04	− 0.026	**0.860**	− 0.042	0.039	− 0.062	0.006	− 0.028	0.040	− 0.088
	SPTT05	− 0.023	**0.859**	− 0.036	0.033	− 0.065	0.009	− 0.032	0.040	− 0.101
	SPTT06	0.035	**0.626**	− 0.092	0.110	0.012	0.010	0.164	− 0.036	0.020
Testing (TST)	TST01	0.000	− 0.028	**0.829**	− 0.077	− 0.027	− 0.036	− 0.014	0.012	0.047
	TST02	0.008	− 0.059	**0.785**	0.080	− 0.009	− 0.072	− 0.107	0.013	− 0.073
	TST03	0.021	− 0.018	**0.779**	0.045	0.021	0.004	− 0.155	0.079	0.009
	TST04	0.036	0.012	**0.752**	0.024	0.089	0.046	0.106	0.018	0.160
	TST05	0.012	0.011	**0.739**	− 0.030	− 0.028	0.014	0.215	− 0.097	− 0.060
	TST06	− 0.032	0.060	**0.709**	− 0.042	− 0.016	− 0.022	0.079	− 0.053	− 0.086
Speaking (SPK)	SPK01	− 0.012	− 0.054	0.014	**0.915**	− 0.019	− 0.018	0.038	− 0.031	− 0.039
	SPK02	− 0.005	− 0.043	0.030	**0.905**	− 0.048	− 0.013	0.031	− 0.030	− 0.054
	SPK03	− 0.040	− 0.002	− 0.026	**0.815**	− 0.012	− 0.062	0.010	0.030	− 0.056
	SPK04	0.028	0.155	− 0.026	**0.680**	0.028	0.097	− 0.153	0.071	0.061
	SPK05	0.022	0.098	− 0.002	**0.655**	0.121	− 0.039	0.032	− 0.024	0.050
Reading (RED)	RED01	0.001	0.020	− 0.037	− 0.003	**0.779**	0.013	0.017	0.026	− 0.092
	RED02	0.079	− 0.068	− 0.007	0.055	**0.743**	− 0.087	0.112	0.112	0.121
	RED03	− 0.019	− 0.047	− 0.061	0.063	**0.742**	0.169	0.037	− 0.028	− 0.006
	RED04	0.031	0.060	− 0.106	− 0.110	**0.709**	− 0.212	0.104	0.116	− 0.066
	RED05	− 0.037	− 0.054	0.054	0.083	**0.693**	0.027	− 0.046	− 0.156	0.047
	RED06	− 0.100	0.115	0.214	− 0.082	**0.654**	0.103	− 0.179	− 0.050	− 0.111
Writing (WRT)	WRT01	− 0.024	0.016	− 0.047	0.032	0.078	**0.775**	0.051	0.063	0.129
	WRT02	− 0.044	− 0.021	− 0.001	0.046	0.044	**0.751**	− 0.079	0.133	0.113
	WRT03	− 0.037	− 0.008	− 0.019	− 0.030	0.041	**0.722**	− 0.049	− 0.020	0.059
	WRT04	0.050	0.011	− 0.006	0.023	− 0.024	**0.708**	0.091	− 0.165	− 0.114
	WRT05	0.069	− 0.008	0.068	− 0.079	− 0.103	**0.706**	− 0.027	0.006	− 0.117
	WRT06	0.013	− 0.009	− 0.075	− 0.046	0.018	**0.684**	0.067	0.029	− 0.109
Student enjoyment (SENJ)	SENJ01	− 0.018	0.024	0.003	− 0.038	0.049	0.037	**0.824**	− 0.003	− 0.027
	SENJ02	0.011	− 0.039	0.022	0.090	− 0.013	0.002	**0.794**	0.005	0.004
	SENJ03	− 0.016	0.078	0.007	− 0.106	− 0.062	0.078	**0.789**	0.022	0.011
	SENJ04	− 0.015	− 0.011	0.002	0.030	0.076	− 0.069	**0.770**	− 0.013	0.004
Listening (LST)	LST01	0.012	− 0.008	0.006	− 0.081	0.056	− 0.055	− 0.065	**0.772**	− 0.130
	LST02	− 0.012	− 0.038	− 0.004	0.019	0.105	0.029	0.025	**0.755**	− 0.031
	LST003	− 0.066	0.055	0.138	0.056	− 0.200	0.047	0.112	**0.692**	0.057
	LST04	− 0.007	0.036	0.063	0.036	− 0.104	0.025	0.102	**0.692**	− 0.013
	LST05	0.082	− 0.037	− 0.143	− 0.002	0.058	0.023	− 0.104	**0.675**	0.009
Student emotional exhaustion (STEX)	STEX01	0.019	0.033	0.123	0.001	0.065	0.025	− 0.103	− 0.023	**0.814**
	STEX02	0.003	− 0.010	0.041	− 0.008	− 0.007	0.011	0.073	− 0.065	**0.776**
	STEX03	0.048	0.024	− 0.126	0.015	− 0.102	− 0.021	− 0.002	− 0.056	**0.737**
	STEX04	− 0.081	− 0.047	− 0.069	− 0.074	− 0.053	− 0.049	0.028	0.026	**0.653**

**Table 4:** Means, standard deviation, and correlations

Variable	M	SD	1	2	3	4	5	6	7	8	9	10	11	12
1.Student perception of teacher talk	3.670	0.900	**0.871**											
2.Student enjoyment	3.630	0.800	0.326**	**0.793**										
3.Student emotional exhaustion	2.440	0.760	− 0.115*	− 0.109*	**0.748**									
4.Student self-efficacy	4.250	0.790	− 0.285**	− 0.480**	− 0.004	**0.830**								
5.Listening	3.980	0.590	0.237**	0.227**	− 0.369**	− 0.173*8	**0.721**							
6.Writing	3.880	0.780	0.049	0.026	0.030	0.029	− 0.050	**0.800**						
7.Reading	3.350	0.700	0.043	0.033	− 0.187**	0.085	0.046	− 0.082	**0.721**					
8.Speaking	4.030	0.600	0.350**	0.118*	− 0.282**	− 0.156**	0.272**	− 0.069	0.081	**0.800**				
9.Testing	3.580	0.770	0.160**	0.399**	− 0.132**	− 0.319**	0.169**	0.000	0.107*	0.231	**0.768**			
10.Education Level	**NA**	**NA**	0.033	− 0.134**	− 0.046	0.106*	− 0.070	0.158**	0.056	0.009	− 0.071	**NA**		
11.Age	**NA**	**NA**	− 0.037	0.109*	0.040	0.084	0.043	0.147**	0.118*	0.138**	0.124*	0.095	**NA**	
12.Gender	**NA**	**NA**	0.064	0.018	0.242**	− 0.177**	− 0.094	− 0.021	− 0.067	− 0.097	− 0.014	0.121**	− 0.055	**NA**

Note: *p < 0.05, **p < 0.01

**Table 5:** CFA approach displaying model comparison outcomes

Models	χ2	df	χ2/df	CFI	TLI	IFI	RMSEA	SRMR
1-factor	10776.28	1313	8.207	0.430	0.402	0.432	0.135	0.186
2-factor	10304.66	1312	7.854	0.458	0.431	0.461	0.133	0.177
3-factor	10266.75	1310	7.837	0.461	0.433	0.463	0.132	0.121
4-factor	7434.095	1307	5.688	0.631	0.611	0.633	0.109	0.102
5-factor	2817.237	1277	2.206	0.906	0.898	0.906	0.055	0.056

Note: 4-factor model: Combined English language attainment, and Student self-efficacy; 3-factor model: Combined English language attainment, Student self-efficacy and Student perception of teacher talk; 2-factor model: Combined English language attainment, Student self-efficacy, Student perception of teacher talk, and student enjoyment

### Assessment of bias (CMB)/ multicollinearity

Due to the cross-sectional survey data limitations like multicollinearity and CMB, we performed several analysis in the present study. Firstly, we conducted a VIF test to diagnose the level of multicollinearity that may exists in the dataset. The VIF scores of all the principal constructs observed (1 to 1.213), indicating that there is no possibility of multicollinearity in this research study. Additionally, the extent of multicollinearity was evaluated using pairwise squared correlation coefficients. As presented in the correlation matrix (Table 4), the squared correlations of constructs are below the recommended value of 0.80, further confirming that multicollinearity is not a concern in this research [73].

Further, to mitigate potential of CMB issues, we implemented several robust techniques as suggested by [74]. To address this concern, several statistical techniques were employed alongside specific measures implemented during the initial stages of the study [74, 75]. In terms of procedural safeguards, beyond the invitation letter, we made efforts to ensure that participants were given sufficient time and space to complete the survey, thus reducing the chance of rushed or incomplete responses. We also included a statement that clarified participants’ rights, including the protection of their personal data and assurance of anonymity. This helped build trust, further reducing the potential for social desirability bias or self-report biases that might arise from participants feeling pressured to respond in a socially acceptable manner. We also emphasized confidentiality and highlighted that their individual responses would remain anonymous and would be aggregated for analysis. This confidentiality assurance aimed to minimize social desirability biases, where participants might otherwise feel compelled to provide responses they believe would be viewed more favorably. For statistical analysis, first, we scrutinized the correlations among all constructs in Table 4. None of the correlation values surpassed 0.90, with the highest being 0.480, indicating the absence of significant CMB [71]. Second, we analyzed the full data set using Harman’s single factor test through exploratory factor analysis. The first factor accounted for only 14.95% of the variance, which is below the 50% threshold. Finally, we compared the fit of the one-factor model to that of the measurement model, as detailed in Table 5. The fit indices for the one-factor model (χ2 = 10776.28 df = 1313, CFI = 0.430, TLI = 0.402, IFI = 0.432, REMSA = 0.135, SRMR = 0.186) were significantly inferior to those of the measurement model (χ2 = 2817.237, df = 1277, CFI = 0.906, TLI = 0.898, IFI = 0.906, REMSA = 0.055, SRMR = 0.056) [76]. Given the assessments made above as well as the statistical test results, it is possible to conclude that CMB does not pose a threat to the research model employed in this study.

### Confirmatory factor analysis

The presented findings of Table 5 indicated the best fit indices for five factor measurement model of the values (χ2 = 2817.237, df = 1277, CFI = 0.906, TLI = 0.898, IFI = 0.906, REMSA = 0.055, SRMR = 0.056) are significantly better than other models. According to Table 5 the value of fit for five-factor model is reasonable when the sample size is above 150 as suggested by [77].

### Hypothesis testing

**Fig. 2:**
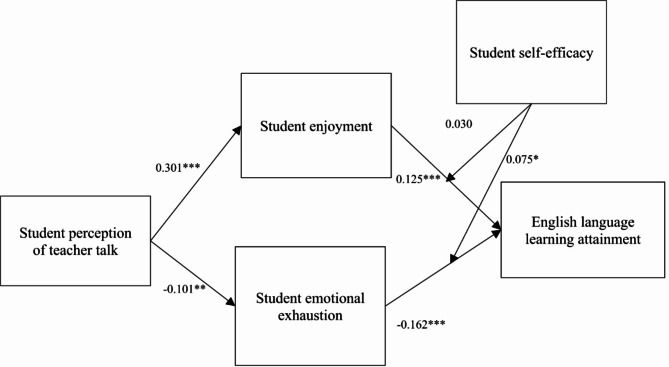
Structural model (Sex = -0.013, Age = 0.077***, Education Level = 0.017). Note: *p < 0.05, **P < 0.01, ***p < 0.001. *Control Variables: Gender*,* Age*,* Education level are insignificant*

With reference to the measurement model’s validity, the formulated hypotheses are examined through a SEM run in AMOS 21. 0 [58]. Furthermore, prior to testing the mediating hypothesis, SEM was used to determine the path among the constructs as shown in Fig. 2. The outcomes have revealed that student perception of teacher talk has a positive connection with student enjoyment (B = 0.301; t = 6.835; *p* < 0.001), and have a negative effect with student emotional exhaustion (B = -0.106; t = -2.298; *p* < 0.01). Student enjoyment (B = 0.125; t = 8.569; *p* < 0.001), and student emotional exhaustion (B = -0.162; t = -7.677; *p* < 0.001) are also significantly related to English language learning attainment. Furthermore, the findings showed that all the control variables were not significantly associated with English language learning attainment expect age.

### Mediation analysis

We also proposed the mediating effects of student enjoyment and student emotional exhaustion. To test these effects, we adopted the bootstrap sampling method (bootstrap sample size = 5000) recommended by MacKinnon, Lockwood [78] to generate asymmetric confidence intervals (CIs) for indirect relationships. Unlike traditional methods such as the Sobel test, the bootstrap CI approach provides a more accurate estimation by producing asymmetric CIs for indirect relationships based on the respective distributions of the two regression coefficients that comprise the product term [78]. Table 6 presents the results of the mediating effects of student enjoyment and student emotional exhaustion. Student enjoyment mediated the relationship between student perception of teacher talk and English language learning attainment, as the CI (LLCI = 0.016; ULCI = 0.056) did not include zero, thus supporting H1. Similarly, student emotional exhaustion mediated the relationship between student perception of teacher talk and English language learning attainment, as the CI (LLCI = 0.006; ULCI = 0.0355) did not include zero, thus supporting H2.

**Table 6:** Mediation analysis

Indirect effect (IV-M-DV)					
IV	M	DV	Coefficient	95% BCCI	Result
Student perception of teacher talk	Student enjoyment	English language learning attainment	0.034	[LLCI = 0.016; ULCI = 0.056]	Supported
Student perception of teacher talk	Student emotional exhaustion	English language learning attainment	0.017	[LLCI = 0.006; ULCI = 0.0355]	Supported

Note(s): IV: Independent variable; M: mediator; DV: Dependent variable

### Moderation analysis

Based on the research model, we incorporated interaction terms to test the moderating effect of student self-efficacy. The results, depicted in Fig. 2, demonstrate that moderation effect of student self-efficacy. The analysis confirmed that the interaction term between student enjoyment and English language learning attainment moderated by student self-efficacy was not significant (B = -0.030; t = 0.634; *p* > 0.05), interaction term is insignificant. In addition, the interaction term between student emotional exhaustion and English language learning attainment (B = 0.075; t= -2.229; *p* < 0.05), is significant.

**Fig. 3:**
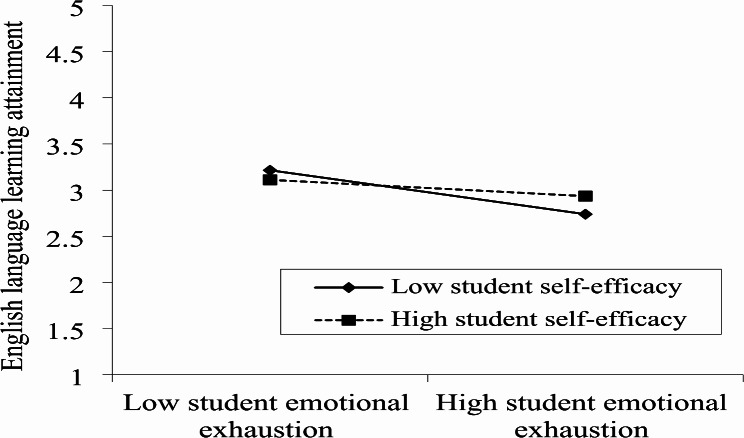
Moderating role of student self-efficacy in the relationship between student emotional exhaustion and english language learning attainment

Furthermore, in order to illustrate the pattern for the moderating role of student self-efficacy, we used a graphical technique as recommended by Aiken, West [79]. In addition, Fig. 3 illustrates that the relationship between student emotional exhaustion and English language learning attainment can be weakened at both high (+ 1SD) and low (-1SD) levels of student self-efficacy.

### Moderated mediation model

We tested the moderated mediation model using SPSS process macro for model 7, as recommended by Hayes [69] for testing the moderated mediation. Table 7 presents the bootstrap confidence intervals (CIs) for the indirect effect of student perception of teacher talk on English language learning attainment through student enjoyment at different levels of student self-efficacy: one standard deviation above the mean (LLC = 0.017, ULC = 0.071), at the mean (LLC = 0.016, ULC = 0.057), and one standard deviation below the mean (LLC = 0.010, ULC = 0.054). The hypothesized mediating indirect effect of student perception of teacher talk on English language learning attainment through student enjoyment was found to be significant because the CIs were not included zero, providing the support for H4. Similarly, Table 7 indicates the indirect effect of student perception of teacher talk on English language learning attainment through Student emotional exhaustion at different levels of student self-efficacy for one standard deviation above the mean (LLC = 0. 008, ULC = 0.053), at the mean (LLC = 0.048, ULC = 0.033), and one standard deviation below the mean (LLC = -0. 020, ULC = 0.033), included zero, H5 is rejected.

**Table 7:** Moderated mediation analysis

**IV= Student perception of teacher talk, Mediator = Student enjoyment, Moderator = Student self-efficacy, DV = English language learning attainment**					
		**Indirect** **Effect**	**SE**	**LLCI**	**ULCI**
Student enjoyment	-1 SD	0.027	0.011	0.010	0.054
Student enjoyment	*M*	0.033	0.010	0.016	0.057
Student enjoyment	*+ 1 SD*	0.038	0.033	0.017	0.071
**IV = Spiritual leadership**,** Mediator = Student emotional exhaustion**,** Moderator = Service climate**,** DV = Employee proactive service performance**					
Student emotional exhaustion	*-1 SD*	0.006	0.013	-0.020	0.033
Student emotional exhaustion	*M*	0.017	0.007	0.048	0.033
Student emotional exhaustion	*+ 1 SD*	0.027	0.011	0.008	0.053

## Discussion

### Implications for theory

The findings of this study extend our theoretical understanding of how students’ perceptions of teacher talk shape their emotional experiences and subsequent language learning outcomes. Specifically, the results suggest that positively perceived teacher talk, characterized by clarity, appeal, and non-threatening delivery, can cultivate enjoyment, which in turn promotes deeper engagement and improved language comprehension and mastery [36, 80]. This positive affective response aligns with the existing theory and research [31, 34], which emphasizes how positive emotions expand cognitive and behavioral repertoires, ultimately fostering more persistent effort and better academic performance [40]. Moreover, the study shows that teacher talk decreases emotional exhaustion, an outcome consistent with the notion that stress depletes cognitive and emotional resources needed for learning [81]. In turn, this emotional fatigue undermines language processing, recall, and overall performance [11, 42]. In turn, this emotional fatigue undermines language processing, recall, and overall performance. Our findings thus validate the central role of emotional exhaustion as a mediating mechanism: although teacher talk directly improves learning, it is the subsequent emotional exhaustion that mediates this impact [48, 82].

Moreover, our study shows that student self-efficacy, a key self-regulated aptitude which measures the level of confidence a student has in his or her capacity to shouldering responsibility and to perform in learning tasks in foreign languages can significantly mediate the effects of enjoyment and emotional exhaustion on language learning [45]. The research study established that high self-efficacy enhanced the impact of enjoyment among the students in the learning of English language compared to the low level of self-efficacy. When students have high self-efficacy they will therefore expend more effort and stay on task longer when faced with difficulties, thus improving their language learning [43, 56]. This finding resonates with Schunk and Pajares [48], who proved that self-efficacious students are also more manageable because they can recover and build on effective learning experiences turning enjoyment to enhanced learning. On the other hand, self-efficacy also plays the role of a moderator which helps to avoid the negative impact of the phenomenon of emotional exhaustion of foreign language learning outcomes. Stressed and fatigued students with high self-efficacy can easily overcome these aspects hence veering the negative impact of emotional exhaustion on cognitive involvement and achievement [43, 45, 56]. Our moderation analysis indicated that treatment effect of emotional exhaustion on English language attainment was significantly less negative among high self-efficacy students compared to those with low self-efficacy, consistent with the moderating effects of self-efficacy posited by Schwarzer and Hallum [83]. This implies that the improvement of self-efficacy could act as a buffer and can help students get back on track in terms of performance, even when they face pressure that comes with emotional stress.

### Implications for practice

From this study, we can derive the following conclusions that can be insightful to educators, policymakers and researchers of language education. Several of these may be highlighted: First is the strong focus on teacher talk as a form of dialogic control that influences the emotional and cognitive participation of students. The more teachers create an interactive classroom environment (e.g., asking open-ended questions, engaging in discussions, using group activities), the more positively students may perceive teacher talk. This approach not only increases students’ enjoyment but also encourages active learning. Specifically, teacher talk that encourages active participation, questioning, and discussion can make students feel more involved in their learning. When students are given opportunities to respond, ask questions, and share opinions, they tend to enjoy the class more. Teachers can ask open-ended questions and facilitate peer interactions to enhance engagement. This study’s implications propose that through professional, enthusiastic, and warm-voiced teacher communications, not only is students’ enjoyment but also their lack of emotional stock diminishes, thus providing the better learning. This highlights the importance for of the professional development programs that helps teachers in proper communication strategies. In essence, with effective strategies and by creating a good environment that teachers cultivate in their classes, the motivation and ability of students to learn is heightened, thus leading to improved language mastery. Further, the moderating influence of student self-efficacy underlines the need to encourage the students, especially through enhancing their self-efficacy in learning the language. Teachers should work with students to identify their individual strengths and encourage them to leverage these strengths in language learning. Teacher can utilize language learning apps, interactive software, and online platforms that offer instant feedback can help students practice and see their progress in real-time. These tools can be motivating and help students develop confidence in their ability to learn at their own pace, fostering a sense of achievement as they use these platforms to improve their language skills. In this case, educators should avoid statements that compromise self-efficacy and instead, practice effective ways of rebuilding it; for instance giving negative feedback, setting realistic goal, and acknowledging small achievements. All the above strategies can help in relieving stress and in making the students strong enough to harness the positive emotions in enhancing of learning.

### Limitations and direction for future research

There are several limitations that should be taken into consideration in our study. A key limitation of this study is the reliance on self-reported measures, which are subject to biases such as social desirability or self-perceived ability bias. Since participants were asked to report on their perceptions of teacher talk, enjoyment, emotional exhaustion, and self-efficacy, their responses may reflect what they believe is socially acceptable or desirable, rather than their actual experiences or feelings. Additionally, students may overestimate their own self-efficacy or downplay negative emotions like exhaustion to present themselves in a more favorable light. This type of bias can undermine the accuracy and validity of the findings, as the data may not fully capture the true emotional or cognitive states of the participants. To mitigate this, future studies could employ more objective measures, such as direct classroom observations or physiological indicators of emotional responses, such as classroom environment or teacher characteristics, on student perceptions and learning outcomes should be addressed, which would provide a more accurate reflection of the students’ emotional and cognitive states during learning.

Furthermore, the cross-sectional design of this study presents a significant limitation in terms of establishing causal relationships. Because data were collected at a single point in time, we cannot definitively conclude that perceptions of teacher talk lead to changes in student enjoyment or emotional exhaustion, nor can we establish how these factors influence language attainment over time. Longitudinal research, which tracks participants over a longer period, would be valuable for determining the directionality and temporal relationships between teacher talk, emotions, and language learning outcomes. For instance, researchers could measure these variables at multiple time points, helping to identify trends and causality, as well as how changes in emotional states or perceptions of teacher talk might influence learning attainment over time.

Furthermore, future studies could benefit from employing a variety of data collection methods, such as direct classroom observations or in-depth interviews, to triangulate the findings. By integrating these additional approaches, researchers can gather richer, more nuanced insights into student perceptions, allowing for a more comprehensive understanding of how teacher talk, emotional responses, and learning outcomes are interconnected. Observational studies could provide a first-hand account of teacher-student interactions, while interviews would offer students an opportunity to express their thoughts and feelings in their own words. This mixed-methods approach would help cross-verify data and reduce potential biases inherent in relying solely on self-reports, leading to more robust and reliable conclusions.

Another significant limitation of this study is its contextual and cultural boundaries. The sample was drawn from a specific university setting, and the findings may not be generalizable to other educational contexts, such as K-12 schools, or to different cultural environments. Students in different regions or educational systems may interpret teacher talk differently, and their emotional responses to the teaching environment could vary widely based on cultural norms and educational practices. For example, cultural attitudes toward authority, communication styles, and student-teacher interactions can all shape how students perceive and respond to teacher talk. Future studies should aim to include more diverse samples across different educational settings and cultural contexts to assess whether the observed patterns hold universally or if they are specific to particular environments. Conducting cross-cultural studies could help determine how teacher talk and students’ emotional responses interact in different cultural contexts, offering a broader understanding of these relationships. The study results could not be applied to all educational environments especially those located in countries where cultural perception regarding interactions between teachers and students and learning of languages are dissimilar. Another direction for future research is to test the results obtained in similar settings to verify the generalizability of the obtained results.

## Data Availability

The datasets used and/or analysed during the current study available from the corresponding author on reasonable request.
